# Equity in practice: Lessons from a cohort-based model for community voice and input in data linkage and use

**DOI:** 10.23889/ijpds.v9i5.2547

**Published:** 2024-09-18

**Authors:** Amy Hawn Nelson, Adelia Jenkins, Deja Kemp, Emily Berkowitz, TC Burnett, Jessie Rios Benitez, Sharon Zanti

**Affiliations:** 1 University of Pennsylvania

## Objective and Approach

Cross-sector data sharing and linkage can transform information about individuals into actionable intelligence to build stronger, healthier, and more just communities. Yet, the use of cross-sector data can also reinforce legacies of racist policies and produce inequitable resource allocation, access, and outcomes. To avoid this, we must embed data equity practices throughout the data life cycle and provide mechanisms for community voice and input. However, few integrated data systems are doing this well.

To combat this challenge, AISP designed a 30-month technical assistance program–the Equity in Practice Learning Community (EiPLC)--to collaboratively develop guidance and models for centering racial equity in data integration. This session provides an overview of our EiPLC Scope & Sequence, a curriculum we designed to guide this work. Currently, 10 jurisdictions in the U.S. are receiving coaching around this curriculum and shifting their data integration practices to advance equity.

## Results

To date, progress has been nonlinear, with positive results. Work in action across the cohorts includes changes to cross-agency data governance, collaborative research agendas, legal agreements, staffing, and community participation practices including.

## Conclusions

While these efforts are nascent, progress is evident and site team members indicate growth as individuals and as site teams. Key features of success include a focus on building relationships, establishing working norms, grappling with racialized histories, and interrogating the role of structural racism and systems of power.

## Implications

The EiPLC provides a successful model for supporting sites to embed data equity principles throughout the data life cycle.

